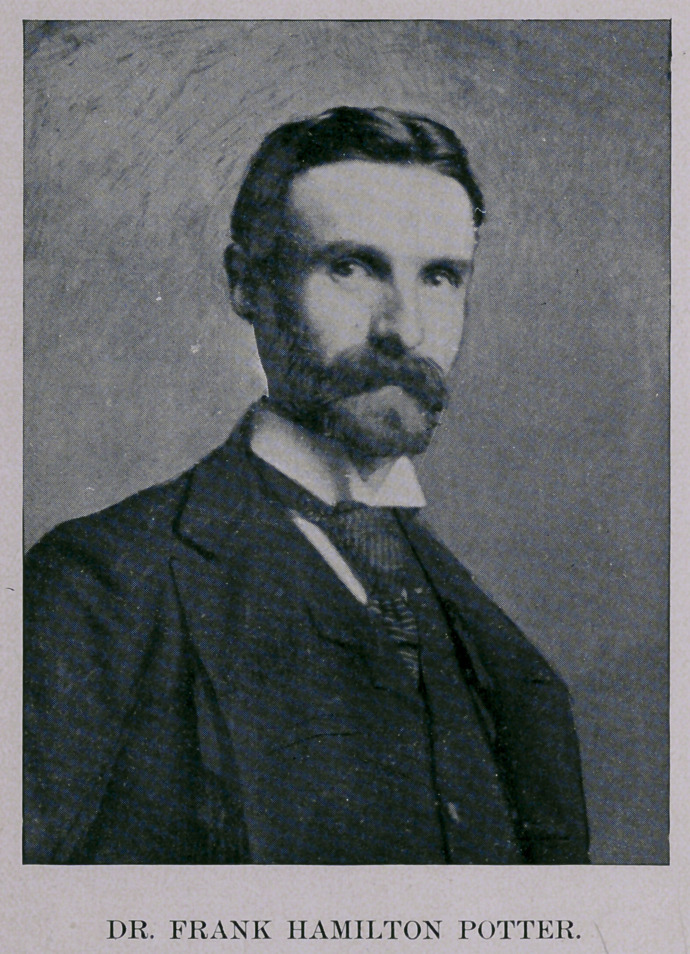# Frank Hamilton Potter, M. D.

**Published:** 1891-08

**Authors:** 


DR. FRANK HAMILTON POTTER.

(*Died July 16, 1891.*)

### BUFFALO MEDICAL AND SURGICAL JOURNAL

A MONTHLY REVIEW OF MEDICINE AND SURGERY.

*EDITORS:*

THOMAS LOTHROP, M. D. - - WM. WARREN POTTER, M. D

All communications, whether of a literary or business character, should be addressed to the editors : 284 Franklin Street, Buffalo, N. Y.

Vol. XXXI. AUGUST, 1891. No. 1.

### FRANK HAMILTON POTTER, M. D.

It is our painful duty to announce to the profession that death has again invaded the editorial corps of the Journal, and taken from the activities of life Dr. Frank Hamilton Potter, for several years an associate editor and co-worker with us. Following within so brief a period the deaths of the lamented Davidson and Campbell, with whom our readers are familiarly acquainted through their contributions to our pages, the sudden taking off of another editorial *confrère*, a brother—beloved for noble qualities of head and heart, and a son who inspired parental pride for his purity of life and conduct, and his honorable ambition in scientific and literary work—is a mysterious dispensation which eternity can alone explain.

The subject of this notice was the only son and eldest child of Dr. William Warren Potter, the managing editor of this journal, and was born in Cowlesville, Wyoming county, N. Y., January 8, 1860. Descended from a long line of American physicians, whose reputation in the State and country has been a source of honorable pride in his family, he early directed his attention to medicine as the profession of his life, and his intellectual training was guided with this end in view. He was graduated at the Buffalo Medical College in the class of 1882, at the early age of twenty-two years. Prior to his graduation, he served in the Rochester City Hospital for two years. After receiving his degree he located in this city, and, on the organization of the Medical Department of Niagara University in 1883, was appointed Clinical Assistant in Surgery. He subsequently held the lectureship of Descriptive Anatomy in 1884, Demonstrator in Surgery, and Lecturer on Botany in

1884–85, Lecturer on Materia Medica from 1885 to 1888, and Lecturer on Laryngology from 1888 to May, 1891. In recognition of his active efforts and conspicuous ability, the Niagara University conferred upon him, in 1885, the *ad eundum* degree of Doctor in Medicine. At the close of the session of 1891, he severed his connection with the school with which from its organization he had labored successfully, and accepted the position of Clinical Professor of Laryngology in the Buffalo University Medical College.

At one time he was a member of the surgical staff of the Sisters of Charity and Emergency Hospitals ; also of the Buffalo Medical and Surgical Association; the Erie County Medical Society; the Buffalo Pathological and Obstetrical Societies ; the Medical Society of the State of New York, and of the American Medical Association, having been Secretary of the Laryngological Section in 1890. He was recently chosen by the Council of the American Laryngological Association to membership in that body — a flattering recognition of his ability and reputation in the special department of medicine which he had selected for his life-work. He was also a member of the Saturn and Thursday Clubs of this city, in which his social qualities and literary attainments were recognized, and his broad and liberal culture highly esteemed by the educated men with whom he delighted to associate.

He was a frequent contributor to the medical and literary societies of which he was a member, the products of his pen commanding attention, both at home and abroad, for their research and clearness of expression as well as beauty of style and diction. The following may be mentioned as some of the titles of his professional contributions :

Treatment of Acute Tonsilitis in Children. B. M. & S. J., Vol. XXVII., p. 455.

Tuberculosis of the Nose, Mouth, and Larynx. B. M. & S. J., Vol. XXVII., p. 295.

Congenital Bony Occlusion of the Nares.— Report of a Case. B. M. & S. J., Vol. XXVIII., p. 74.

Treatment of Acute Coryza. B. M. & S. J., Vol. XXVIII., p. 302.

The Influence of Oral Irritation in the Production of Disease of the Upper Air-Tract. B. M. & S. J., Vol. XXIX., p. 12.

The Use of Menthol in Diseases of the Upper Air-Passages. Journal A. M. A., February 1, 1890.

On the Treatment of Hay-Fever. B. M. & S. J., Vol. XXX., p. 88.

Neurasthenia and Nasal Disease. Loc. Cit. p. 337.

Croupous Rhinitis. (Two papers.) Trans. Med. Soc. S. N. Y., 1889 and 1891.

Cystoma of the Nasal Passages: with Report of a Case. Candidate's Thesis for Membership in American Laryngological Association. (Unpublished.)

Some After-Effects of the Recent Influenza. Read at the meeting of Central New York Medical Association, June 2, 1891. (To be published in BUFFALO MEDICAL AND SURGICAL JOURNAL, September, 1891.)

Among the instruments he devised may be mentioned:

Mechanical Nasal Saw.

Self-retaining Nasal Speculum.

Nasal Scissors.

This too brief review of his professional and literary labors shows how earnestly and faithfully he must have worked to attain, at an early age, such an enviable position in the scientific, literary, and social circles in which he moved, and also with what honor and pride he wore the mantle in medicine bequeathed to him by his progenitors.

Personally, Frank Hamilton Potter was a most genial and courteous gentleman, positive in his principles and convictions, but with an agreeable manner of expressing them, which carried weight without giving offense to his associates. His life was as pure and guileless as that of a child. Whether as son or brother, husband or father, he filled the full measure of duty, conscientiously demonstrating his loyalty to principle, his devotion to family and friends, his ambition to excel when excellence could be obtained by honorable means, his aim to combine a commendable zeal for his profession with the equally imperative responsibilities of citizenship, for which his services were sought.

In 1887, after returning from Europe, whither he went for study and travel, he married Eva, daughter of Lars G. Sellstedt, the famous artist of this city, and two sons were born as a result of this union. The widow and these children survive.

The profession join in the general sorrow pervading the community, and tender their sincere sympathy to the afflicted families for this strange dispensation which robs them of a devoted son and brother, a loving husband and father, and the city of a respected and honorable citizen. T. L.

## Figures and Tables

**Figure f1:**